# T-Cell Response to Hepatitis B Core Antigen: Identification of Prior Exposure to and Confirmatory Testing for Screening for Anti-HBc

**DOI:** 10.1155/2013/812170

**Published:** 2013-12-03

**Authors:** Patricia Araujo, Roger Y. Dodd, Flavia Latinni, Renata Souza, Ricardo Diaz, Jose Augusto Barreto

**Affiliations:** ^1^Infection Diseases Laboratory, Colsan/UNIFESP, Avenida Indianópolis, 1260 Indianópolis, São Paulo, SP, Brazil; ^2^American Red Cross, USA; ^3^Colsan, Technical and Research Department, Brazil; ^4^Colsan, Quality Laboratory, Brazil; ^5^Retrovirology Laboratory, UNIFESP, Brazil; ^6^Colsan, Associação Beneficente de Coleta de Sangue, Head Colsan, Brazil

## Abstract

*Background*. During routine donor screening in the blood bank, it is not uncommon to find isolated reactivity for anti-HBc in the absence of detectable HBV DNA in a first donation but absence of reactivity to anti-HBc in subsequent donations, suggesting a false-positive result for anti-HBc. *Study Design and Methods*. The blood donor population was screened between January 2010 and October 2011. We selected 2,126 donations positive only for anti-HBc from a total of 125,068 donations. During the process, OBI donors were identified, and their HBcAg-specific T-cell response was analyzed and compared to donors with chronic (HBsAg positive) and recovered (anti-HBc only) infection. We analyzed correlations between signal levels (Co/s) in the competitive assay for anti-HBc and HBV DNA detection. *Results*. In the 21-month study period, 21 blood donors with anti-HBc alone were identified as OBI (1 in each 5955 donors). The relevant finding was the observation that anti-HBc only subjects with Co/s ≥ 0.1 did not have either HBcAg-specific T-cells or detectable HBV DNA and OBI subjects presented with Co/s ≤ 0.1 and HBcAg T-cell response. In the subset of 21 OBI subjects, 9 donors remained positive for HBcAg T-cell response after four collections. In all 9 samples, we observed HBV DNA fluctuation. *Conclusion*. Our data suggest that HBcAg-specific T-cell response could be used to confirm anti-HBc serological status, distinguishing previous exposure to Hepatitis B virus from anti-HBc false-positive results.

## 1. Introduction

Hepatitis B virus (HBV) infection is an important global health problem [1, 2] especially in Asia, Africa, Southern Europe, and Latin America [3]. About 2 billion people are infected with HBV worldwide [4–7], among them, 400 million suffer chronic HBV infection [5]. In Brazil, the HBsAg positivity ranges from 1.6% to 8.5%, corresponding to approximately 3 to 16 million infected individuals [8–13]. The residual HBV risk in screened blood transfusion ranges from 1 : 10,700 [14] to 1 : 62,734 [15] and is markedly higher compared to Europe [16], North America [17, 18], Australia [19], and Japan [20].

Further, the identification of blood donors with occult HBV infection (OBI) individuals negative for Hepatitis B antigen (HBsAg) with detectable circulating HBV DNA has created concern related to the safety of the blood supply [21, 22].

Hepatitis B virus continues to offer the greatest risk of transfusion-transmission infection despite HBsAg screening of blood donations. Residual risk of HBV transfusion-transmission results from occult HBV infection, window period donations, and possible escape variants. There is a need to develop cost-effective algorithms using available serological and NAT methods to increase blood safety without compromising the availability of blood.

False-positive results in anti-HBc testing and false-negative results in NAT methods can hamper the identification of occult HBV infection. In anti-HBc testing, false-positive result to anti-HBc are attributed to nonspecific reactions associated with competitive anti-HBc enzyme immunoassay, possiblly due to IgA or IgM-related molecules produced from nonspecifically activated HBV-specific B lymphocytes or cross-reactivity with other serum components [22]. In donor testing using NAT pooling methods, the low viral load found in most OBIs may affect blood safety and screening algorithms in which confirmation depends upon DNA detection.

The reasons for low HBV DNA levels in absence of detectable HBsAg in OBI remain undefined, but it is conjectured that both host and viral factors are important in suppressing viral replication and maintaining control of the infection [5, 6, 23].

T-cell mediated responses contribute to the control of viral replication, protect against reinfection, and may lead to spontaneous resolution of infection in OBI [24]. Patients who successfully clear the virus present a T-cell response to HBsAg, core and polymerase proteins [25, 26]. Additionally, increased HBcAg- and HBeAg-specific T-helper cell responses are also seen in patients with viral clearance [27].

The T-cell response in individuals with an anti-HBc only profile showed a typical protective memory when compared to anti-HBc-negative blood donors that have not been exposed to HBV. Cellular and humoral immune response pressure against HBV envelope proteins are the principal mechanisms originating OBI [28].

In this context, the aim of this study was to develop algorithms/methods to confirm anti-HBc test results. A new method is proposed: detection of HBcAg-specific T-cell responses.

## 2. Materials and Methods

### 2.1. Blood Donors Included for Cellular Immunity Assessment

The blood donor population from COLSAN-Associação Beneficente de Coleta de Sangue (São Paulo, Brazil) was tested between January 2010 and October 2011. We selected 2,126 donors found positive only for anti-HBc from a total of 125,068 donations. During the process, OBI donors were identified, and their HBcAg-specific T-cell response was analyzed and compared to donors with chronic (HBsAg positive) and recovered (anti-HBc only) infection. As control subjects, we enrolled 100 healthy blood donors that had no reactivity for HBV including HBV PCR, and who were nonreactive for Hepatitis C Virus (HCV), Human Immunodeficiency Virus (HIV), Human T-lymphotropic virus (HTLV), Syphilis, and Chagas Diseases. All subjects gave their informed consent, and the study was approved by the UNIFESP Ethical Committee.

### 2.2. Detection of Serological HBV Markers

Blood units collected at COLSAN-Associação Beneficente de Coleta de Sangue (São Paulo, Brazil) were screened for the presence of serum anti-HBc and HBsAg using a commercial enzyme linked immune-assay (ELISA) (BioMérieux, France). HBe, anti-HBe, anti-HBs, and Core IgM testing was performed using the ELFA system (BioMéreiux, France). HBsAg was confirmed by neutralization test (BioMérieux, France). All the samples were also screened by ELISA for possible HCV, HTLV, HIV infections, syphilis, and Chagas Diseases (ELISA) (BioMérieux, France). Samples with additional reactivity for any other infection were excluded from this study.

### 2.3. HBV DNA Detection

Serum samples were stored at –20°C and thawed immediately before use. For detection of HBV DNA, DNA was extracted and subsequently subjected to a commercial test for HBV-DNA (COBAS AmpliPrep/COBAS TaqMan HBV Test, v2.0) with a range from as low as 20 IU/mL to as high as 1.7 E + 08 IU/mL.

### 2.4. Peripheral Blood Mononuclear Cells Preparation

Peripheral blood mononuclear cells (PBMCs) were freshly isolated from heparinized blood by Ficoll-Hypaque density gradient centrifugation with Lymphoprep (Axis-Shield, Oslo, Norway) as previously described [23]. Subsequently, cells were resuspended in complete RPMI-1640 Medium (Gibco, Invitrogen, Beijing, China), which contained 10% heat-inactivated fetal bovine serum (Gibco, Invitrogen, Australia), 2 mM L-glutamine, 100 U/mL penicillin, and 100 *μ*g/mL streptomycin.

### 2.5. HBcAg Specific T-Cell-Response

T-cells were cultured at 10^6^ cells per mL in RPMI-1640 (Gibco, Invitrogen, Beijing, China) supplemented with 10% heat-inactivated human AB serum, 2 mmol/L l-glutamine, 100 U/mL penicillin, and 100 *μ*g/mL streptomycin and incubated overnight at 37°C and 5 percent CO_2_. Recombinant HBcAg (rHBcAg) expressed in *Escherichia coli* are based on cloned HBV DNA of HBV genotypes A, B, C, D, and F was purchased from DiaSorin, Saluggia, Italy. Seventeen overlapping 20-mer peptides covering the entire HBV core sequence (amino acids 1 to 183) were synthesized with a multiple-peptide synthesizer using standard 9-fluorenylmethoxy carbonyl chemistry (Syro, MultiSynTech, Bochum, Germany). Recombinant-HBcAg (10 *μ*g/mL) was added, and cells were further incubated for 5 days. Control cultures included 2 *μ*g/mL phytohemagglutinin (PHA), medium alone, and recombinant HCV NS5 protein, (American Research Products, Belmont, MA). [3H]-Thymidine (specific activity 5 mCi/mmol) was added for the last 20 hours of culture. Results were expressed as counts per minute (cpm) for PHA-stimulated cultures and as stimulation index (SI), for example, the ratio between median cpm in the HBcAg-containing cultures and median cpm of control HCV NS5-containing cultures. The 99th percentile of HBcAg-specific SI in 10 healthy blood donors was 2.01. For practical purposes, HBcAg-specific T-cell response was scored positive when SI > 3.

### 2.6. Enzyme-Linked Immunospot Assay (ELISpot) for Interferon-Gamma (IFN-*γ*)

The antigen used for the human IFN-*γ* ELISpot assays was the same used in HBcAg T-cell response (rHBcAg). Multiscreen-IP 96-well plates (Millipore, Billerica, MA) were coated overnight at 4°C with 10 *μ*g/mL anti-human IFN-*γ* monoclonal antibody (1-DIK; Mabtech, Sweden). Plates were washed seven times with DPBS and blocked with RPMI 1640 supplemented with 10% fetal bovine serum for 2 hours at room temperature. Freshly isolated or frozen-thawed PBMCs (2.5 × 10^5^/well) were seeded in duplicates. Plates were incubated for 32 hours at 37°C with 5% CO_2_. After washing, 50 *μ*L of 1 *μ*g/mL biotinylated monoclonal antibody to INF-*γ* (7-B6-1; Mabtech, Sweden) was added to each well and incubated for 2 hours at room temperature; plates were subsequently washed seven times and 50 *μ*L per well of 1 *μ*g/mL Streptavidin-Alkaline phosphatase (Mabtech, Sweden) was added, and plates were further incubated for 1 hour at room temperature. Plates were washed seven times, and 100 *μ*L per well of BCIP/NBT (diluted by distilled water; Zymed BCIP/NBT SUBSTRATE KIT, Invitrogen, Camarillo, CA) was added. After 10 minutes, the colorimetric reaction was stopped by distilled water and washed three times with distilled water. Plates were air dried, and the ImmunoSpot S4 Macro Analyzer (Cellular Technology Ltd, USA) was used for spot counting. Results were expressed as numbers of spot-forming cells (SFC) per 10^6^ PBMCs. The number of specific IFN-*γ*-secreting cells was calculated by subtracting the value of the unstimulated control from the value of the stimulated sample. The positive control consisted of PBMCs stimulated with 10 *μ*g/mL of PHA, and the criteria for a positive response for the ex vivo ELISpot assays were more than 5 SFC per well and more than twice the number of SFC than the unstipulated control well. Therefore, in this study, more than 20 SFC per 10^6^ PBMCs would be a positive response.

### 2.7. Statistical Analysis

Comparison of paired data from patients at different times of followup was performed by Wilcoxon's signed-rank test. Data from groups of unrelated subjects were compared by Mann-Whitney *U* test. Correlation between HBV DNA and HBc-specific SI was assessed by Spearman's *ρ* test.

## 3. Results

### 3.1. Serological HBV Screening and HBV DNA Detection

**Figure 1:**
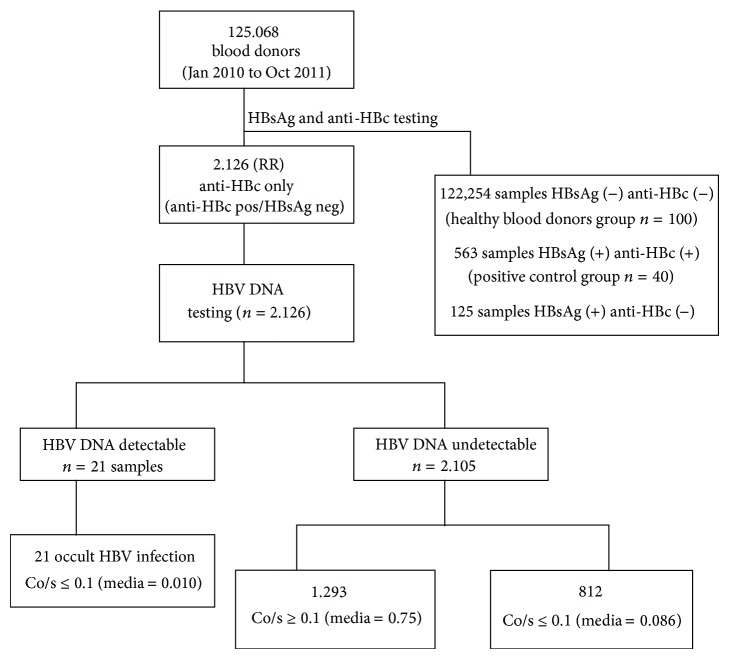
Algorithm for classification of studied samples. Co/s = relation between optical density and cutoff in competition assay for anti-HBc (BioMerieux). Among 563 samples with HBsAg (+) anti-HBc (+), 438 samples had HBV DNA detectable. Among 125 samples HBsAg (+) andti-HBc (−), 27 samples had HBV DNA detectable.

A total of 2,126 of 125,068 donations, (1.7%) were positive for anti-HBc, and none of them had detectable HBsAg in the serum. Reactivity only HBsAg was found in 125 (0.1%) donations, and HBsAg plus anti-HBc was observed in 563 (0.45%) donations. Both HBsAg and anti-HBc were absent in 122,254 (97.75%). Among 2,126 (HBsAg−/anti-HBc+) only 21 samples had detectable HBV DNA. Among the 125 samples with primary infection (HBsAg+/anti-HBc−), 27 samples had detectable HBV DNA. Among 563 samples with chronic infection (HBsAg+/anti-HBc+), 438 had detectable HBV DNA (Figure 1). Between January 2010 and July 2010, when HBV DNA screening was performed using minipools of 6 samples, we did not observe positive samples in a total of 12.000 screened samples from blood donors with HBsAg and anti-HBc negative. In the 21-month study period, 21 blood donors with anti-HBc alone profile were identified as OBI (1 in each 5955 donors). In the same period, the number of blood donors with reactivity for HBsAg and anti-HBc was 113 (1 in each 1106 blood donors).

### 3.2. Signal Values (Co/s) for Anti-HBc in Competition Assay

Among the group of 2,126 samples with anti-HBc only, test reactivity (represented as Co/s) ranged between 0.008 and 0,975. The samples were divided in two groups based on a threshold of Co/s = 0.1 and further tested for HBV DNA. 21 samples had HBV DNA detectable and Co/s ≤ 0.1 and then was classified as OBI, and 2,105 anti-HBc only segregating into 1,293 anti-HBc Co/s ≥ 0.1 and 812 Co/s < 0.1 (Figure 1).

### 3.3. HBcAg-Specific T-Cell Response

**Figure 2:**
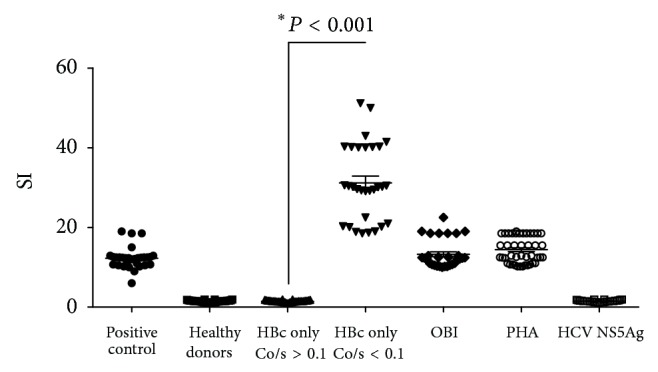
The proliferative response of peripheral blood lymphocytes to nonspecific (PHA) and specific stimuli (HBV core antigen; HCV NS5 antigen) in studied groups. SI = index of stimulation. HBc only with Co/s ≥ 0.1: absence of T-cell response and HBV DNA classified as false-positive to anti-HBc. HBc only with Co/s ≤ 0.1: presence of T-cell response and HBV DNA classified as OBI. HBc only with Co/s ≤ 0.1: presence of T-cell response and HBV DNA undetectable classified as spontaneous HBV resolvers.

**Figure 3:**
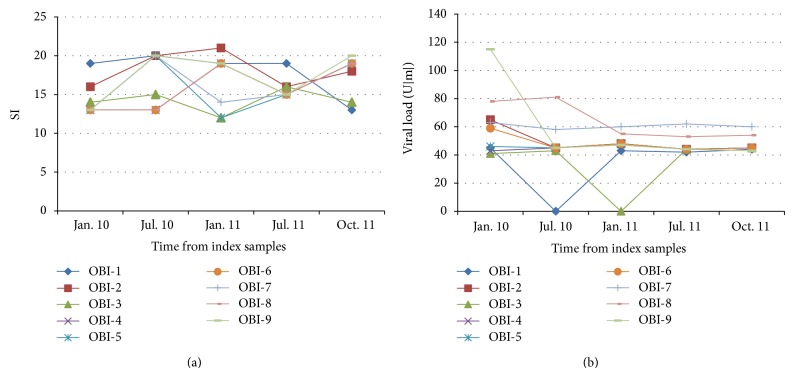
HBcAg T-cell response and HBV load in 9 cases of OBI. OBI-1 to OBI-9 are related to samples with occult HBV infection.

The specificity of the anti-HBc response in these two groups of anti-HBc only samples was investigated further by analyzing the HBcAg T-cell response through T lymphocyte proliferation and INF-*γ* response to specific and nonspecific stimuli. The proliferative response of peripheral blood lymphocytes to nonspecific (PHA) and specific stimuli (HBV core antigen; HCV NS5 antigen) was analyzed in HBV carriers, OBI, healthy donors, and two groups based on a threshold of Co/s ≤ 0.1 and Co/s ≥ 0.1. The relevant finding was the observation that anti-HBc only subjects with Co/s ≥ 0.1 (*n* = 1.293) did not have either HBcAg-specific T-cells or detectable HBV DNA in any analyzed samples. These subjects were classified as “false-positives” for anti-HBc, corroborated by the absence of reactivity to anti-HBc in the subsequent donations. The median signal value obtained in the “false-positive” group was equal to healthy blood donors (SI < 3,0). All anti-HBc only with Co/s ≤ 0.1 (*n* = 833) presented HBcAg-specific T-cell responses; however, 21 out of 833 presented detectable HBV DNA (OBI). The median SI obtained in HBV carriers and the OBI group was 13,6 and 12,2, respectively. The subjects (*n* = 812) with Co/s ≤ 0.1 and HBcAg T-cell response and undetectable HBV DNA are classified as spontaneous HBV resolvers. Spontaneous HBV resolvers showed a large peripheral blood mononuclear cell (PBMC) response (SI = 36,2) to HBcAg when compared to HBV carriers and OBI (SI = 12,2) (*P* < 0.001) (Figure 2). In the subset of 21 OBI subjects, 9 collected samples viral load was quantified in all donations and fluctuated between <10 and 118 IU/mL, whereas in three samples HBV DNA was not detected, but HBcAg T-cell response remained positive in all the study period (Figure 3).

### 3.4. Enzyme-Linked Immunospot Assay (ELISpot) for IFN-*γ*

**Figure 4:**
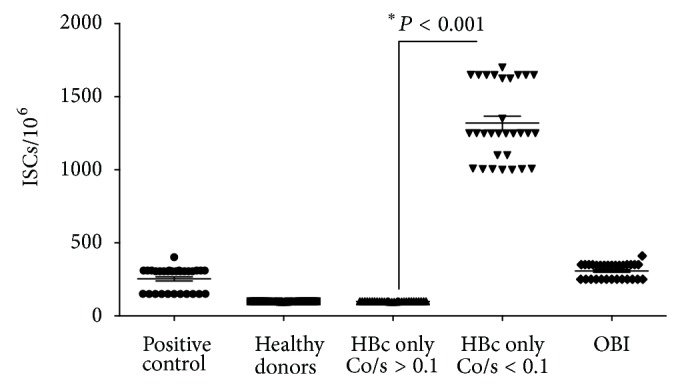
ELISpot-INF-*γ* assays in studied groups. ISCs: index spot-forming cells index. HBc only with Co/s ≥ 0.1: absence of T-cell response and HBV DNA classified as false-positive to anti-HBc. HBc only with Co/s ≤ 0.1: presence of T-cell response and HBV DNA classified as OBI. HBc only with Co/s ≤ 0.1: presence of T-cell response and HBV DNA undetectable classified as spontaneous HBV resolvers.

ELISpot-INF-*γ* assays were positive in HBc only with Co/s ≤ 0.1, OBI, and positive control. The magnitude of T-cell responses to HBcAg in HBc only with Co/s ≤ 0.1 ranged from 1,020 to 1,628 ISCs/10^6^ PBMCs, whereas among OBI ranged from 132 to 548 ISCs/10^6^ PBMCs, among positive controls from 160 to 582 ISCs/10^6^ PBMCs, and among HBc only Co/s > 0.1 and healthy blood donors from 8 to 15 ISCs/10^6^ PBMCs. The frequency of positive IFN-*γ* ELISpot responses in spontaneous HBV resolvers indicated a higher chance to have demonstrable HBV-specific T-cell responses than among positive controls (*P* < 0.001) and among OBI (*P* < 0.001) (Figure 4).

### 3.5. Serological Markers for HBV in OBI and Spontaneous Resolvers Blood Donors

**Figure 5:**
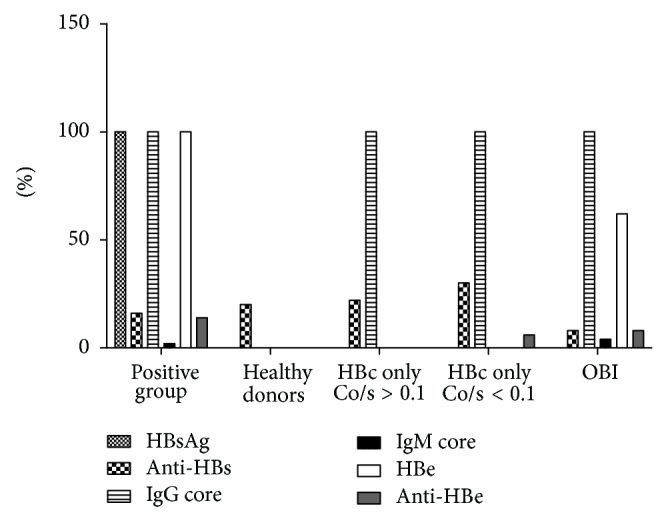
Serological markers from HBV in studied groups. HBc only with Co/s ≥ 0.1: absence of T-cell response and HBV DNA classified as false-positive to anti-HBc. HBc only with Co/s ≤ 0.1: presence of T-cell response and HBV DNA classified as OBI. HBc only with Co/s ≤ 0.1: presence of T-cell response and HBV DNA undetectable classified as spontaneous HBV resolvers.

Serological markers of HBV exposure or vaccination (anti-HBs) were observed in 9.1% (3/40) from primary infection (HBV carriers); 21% (21/100) of healthy donors; 23,5% (303/1293) of HBc only with Co/s ≥ 0.1; 32.4% (263/812) of HBc only with Co/s ≤ 0.1; and 10,2% in OBI. Reactivity to IgM anti-core was observed in 2.5% (1/40) and 5.0% (2/21) of HBV carriers and OBI, respectively. HBe antigen was observed in all blood donors with OBI and in HBV carriers but not in healthy donors and HBc only. However, 10.5% (2/21) of OBI and 12.5% of (5/40) HBV carriers showed anti-HBe-positive. In healthy donors and HBc only, no reactivity to anti-HBe and Core IgM was observed (Figure 5).

## 4. Discussion

Hepatitis B virus presents a higher residual risk of transmission by transfusion than hepatitis C virus (HCV) or human immunodeficiency virus (HIV), especially in countries of South America, like Brazil, where the prevalence of HBsAg in the general population ranges from 2 to 20% [22]. In this study we found HBV DNA detectable in 21.6% of blood donors with primary infection (HBsAg (+) anti-HBc (−) and 77.8% of chronic blood donors (HBsAg (+) anti-HBc (+)). These data are consistent with the observation by several authors about HBV persistence in chronic infection [29, 30].

The persistence of HBV DNA was observed like OBI in 0.98% of the 1.7% of blood donors which were anti-HBc positive, corroborating results of previous studies [31] that observed 3.3% OBI subjects into of 5.6% of blood donors with anti-HBc alone profile. The same assays for detection of HBsAg and anti-HBc were used in both studies, and the detection limit in HBV DNA PCR was similar. In the general blood donor population, the prevalence of OBI in this study was 1 in 5,955 (0.017%), and, in the same period, HBV carrier rate was 1 in 1,106 blood donors. The OBI among blood donors varies from country to country in different parts of the world. For example, in the European countries such as Poland, Italy, Spain, and Germany, the OBI prevalence rates of 0.006%, 0.22%, 0.05%, and 0.0006%, respectively, have been reported by Candotti et al. [32]. In addition, it seems that the prevalence of OBI in blood donors has a regional variance, not only internationally but also nationally. This national discrepancy can be observed in Brazil, where HBV prevalence differs regionally, varying from 2–7% in the north east to 2–75.6% in north west [33].

In this context, testing for anti-HBc is important, principally in the detection of cases where HBsAg is negative and HBV DNA detectable (OBI). However, a high frequency of reactivity to anti-HBc is observed in Brazil ranging from 1.0% to 5.0% in Northeast, Midwest, Southeast, and South of Brazil but can reach alarming levels of 75.6% in the Amazonia region. In this study, we observed a 1.7% of frequency of reactivity to anti-HBc in the absence of HBsAg, according with frequency found in Southeast of Brazil. However, the presence of HBV DNA observed was low representing only 0.98% of the 1.7% of anti-HBc only blood donors. These data raise the question “What is the meaning of anti-HBc reactivity in absence of detectable HBV DNA in blood donors?”

In the serological routine in blood bank, it is not uncommon to find isolated reactivity for anti-HBc and HBV DNA undetectable in first donation followed by nonreactive anti-HBc results in the next donation, suggesting that the initial result was false-positive.

The determination of the frequency of true anti-HBc only in blood donors is very important to blood safety strategy and to indentify recovered infection, escape mutants, and OBI. Algorithms including multiple successive assays are necessary but not sufficient to identify true reactivity to anti-HBc [34]. To overcome this difficulty, Japanese blood banks have set up an anti-HBc cutoff agglutination title of ≥32 and, below this level, donations are considered noninfectious (presumably false-positive or with undetectable anti-HBs) [35]. Actually Japanese blood bank defers all donors with any reactive result for anti-HBc.

In this study we had relevant findings about the correlation between threshold (Co/s) values in anti-HBc competition assay and HBV DNA detection. Among anti-HBc only subjects with detectable HBV DNA, all had Co/s ≤ 0,1. However, among blood donors with Co/s > 0.1, none had detectable HBV DNA. Distinguishing in this group between HBV exposure and false-positive to anti-HBc appears to be defined by the HBcAg T-cell response. We found that, among the anti-HBc only group with Co/s < 0.1, and in the presence of HBV DNA, all donors had a response for HBcAg T-cell in all subjects that we classified as occult HBV infection. In anti-HBc only Co/s > 0.1 and in the absence of HBV DNA when there was an HBcAg T-cell response, we classified these individuals as having spontaneous HBV clearance, and in absence of HBcAg T-cell response we classified them as false-positive to anti-HBc.

These findings are corroborated by the difference in magnitude of HBcAg T-cell response between the groups (HBV carriers and healthy groups), OBI subjects with serial collected samples and ELISpot-INF-*γ* data.

We found a large HBcAg T-cell response for spontaneous HBV clearance, positive controls, and OBI, all subjects with previous HBV exposure when compared healthy group. We observed HBV DNA fluctuation in 9 cases of OBI with serial collected samples; however, the presence of HBcAg T-cell response was observed in all serially collected samples. In the follow-up of cases of OBI, we observed fluctuation of viral load but the HBcAg T-cell response was always positive. This is very important to the identification of OBI because it eliminates several problems that are due to false results in HBV DNA detection, like viral load, assay sensitivity, fluctuation of viral load, repeat of the HBV DNA detection, and other factors (mutant, variants, mosaic, and quasispecies). In the other 11 remaining OBIs, we observed linear HBV DNA detection in two subsequent donations. These blood donors will be followed for more two donations, and the results will be analyzed.

In transfusion medicine, assays to detect anti-HBc is recommended to detect potentially infectious blood donors as OBI. Current guide lines suggest retesting of these samples by alternative assays. However, current commercial anti-HBc assays often generate divergent results. HBcAg specific T-cell response showed high sensibility in distinguishing “true positive” for “false-positive” to anti-HBc.

The results observed in HBcAg- T-cell response corroborate with ELISpot-INF-*γ* data. We observed that spontaneous HBV resolvers presented higher frequency of positivity in this assay when compared to HBV carrier, healthy donors, anti-HBc false-positive results, and OBI (*P* > 0.001). In addition, the magnitude of T-cell responses to HBcAg in HBV spontaneous resolvers was clearly higher when compared to HBV carriers, healthy donors, anti-HBc false-positive results, and OBI (*P* > 0.001). These data suggest that antibody titers indeed correlated with HBcAg- T-cell response and ELISpot-INF-*γ*.

The presence of immune response was found in ELISpot-INF-*γ* in spontaneous HBV resolvers, and OBI and HBV carriers are according to findings of Zerbini et al. who reported that the presence of memory T-cell response in anti-HBc positive individuals with OBI could be important for maintaining viremia under control but persistent [28].

Despite all the evidence pointing to efficiency of HBcAg T-cell response to confirm anti-HBc result in the competition assay, the discrepancy of HBcAg T-cell response in the OBI and positive groups when compared to findings of Zerbini and cols in 2008 and Bes and cols in 2012 was an important point to elucidate. We suggest that the discrepancy between our results and these researchers could be principally due to process of selection of study groups, because if we introduce the group classified for us as false-positive to anti-HBc, the magnitude of HBcAg T-cell response is equal between OBI and positive control, if it is considered to be the median of stimulation index found in true positive and false-positive to anti-HBc, agreeing with Zerbini and Bes studies.

HBcAg T-cell response proved to be an important test to eliminate false-positive results for anti-HBc and that could be used in the research and in blood bank routine. In Brazil, the cost of discarding anti-HBc false-positive donation is high, and the cost to use HBcAg T-cell response in the laboratory that had previous laminar flow cabinet and CO_2_ incubator is low, five times smaller than the cost of false-positive donation. We cannot forget the stigma in the donor to receive a result with positive serology and indirect cost for the public health service to conduct additional tests in order to confirm the positive anti-HBc result. The presence of HBcAg T-cell response in the absence of HBV DNA in cases of OBI suggests that HBcAg T-cell response could be a complementary or in specific cases an alternative to NAT.

Even with detecting other serological markers of hepatitis B like Core IgM, HBeAg, anti-HBe and anti-HBs, the identification of OBI in anti-HBc only is difficult.

Firstly, we investigated the concentration of anti-HBs in OBI, spontaneous HBV resolvers, and HBV carrier, and we observed that frequency was 10.2%, 32,4%, and 9.1%, respectively. Several studies showed that some individuals with OBI or HBV, who have recovered from HBV infection, produce neutralizing anti-HBs. In these same individuals we may observe continuous HBV DNA replication at low levels that are detectable for years in the liver, peripheral blood mononuclear cells or serum [32, 33, 36–39]. The frequency of anti-HBs found in spontaneous HBV resolvers was higher than HBV carriers and OBI probably due to important immunity role of anti-HBs in protection against HBV infection. The presence of anti-HBs in OBI has been reported by many authors with frequency varying from 0.5% to 15% still tested positive for serum HBV DNA, through at a very low titer which is in accordance according with our findings in this study.

Another serological marker studied was anti-HBe, an important marker of HBV chronic carriage [5]. We observed in OBI (10.5%) and HBV carrier (12.9%) the presence of anti-HBe in lower concentration when compared to levels of anti-HBc. This data could be explained by the variable proportion of samples with HBc alone profile, in which anti-HBe is detected. Although, with lower titers of anti-HBe compared to anti-HBc, there comes a point when the former is no longer detectable, leaving the latter as the only serological marker of infection [5]. HBeAg was observed in all blood donors with viremia, including OBI and HBV carrier.

IgM anti-HBc is the first antibody detectable in acute HBV infection, which is usually detected within 1 month after appearance of HBsAg. The presence of IgM anti-HBc with high index value usually indicates a recent HBV infection, and this antibody usually disappears within 6 months. However, the index value of IgM anti-HBc may increase to levels usually detectable in acute infection in 10–20% of chronic hepatitis B patients with acute exacerbation or hepatitis flare that often leads to a misdiagnosis of acute hepatitis B [40]. In our study we observed that the frequency of high index value of IgM anti-HBc was 5.0% in OBI and 2.5% in HBV carrier, suggesting a recent HBV infection.

Our data suggest that HBcAg-specific T-cell response could be used to confirm anti-HBc serological status distinguish previous exposure to Hepatitis B virus and anti-HBc false-positive.
